# Supplementary material for: A multidimensional functional fitness score has a stronger association with type 2 diabetes than obesity parameters in cross sectional data
Source: PLoS One. 2021 Feb 5;16(2):e0245093. doi: 10.1371/journal.pone.0245093 (PMC7864668; doi:10.1371/journal.pone.0245093)
Supplement: Attachment — Submitted filename: response to reviewers.docx [file pone.0245093.s003.docx]

2. Thank you for including your ethics statement:

'The study proposal was reviewed by the Institutional Ethics Committee, In-house
Research, of the Deenanath Mangeshkar Hospital and Research Centre on 27th Aug
2018. Owing to the retrospective nature, the requirement for informed consent was
waived by the committee and the data were anonymized before availing for analysis
which was done between October to December 2018.'

a. Please amend your current ethics statement to confirm that your named institutional review board or ethics committee specifically approved this study.

b. Once you have amended this/these statement(s) in the Methods section of the manuscript, please add the same text to the “Ethics Statement” field of the submission form (via “Edit Submission”).For additional information about PLOS ONE ethical requirements for human subjects research, please refer to <http://journals.plos.org/plosone/s/submission-guidelines#loc-human-subjects-research>'

The ethics statement is modified accordingly and corresponding change in the form made.

3. Thank you for stating the following in the Acknowledgments Section of your manuscript:

"We thank the management of Deenanath Man geshkar Hospital and Research Center that supports the BILD clinic, Mr. Deepak Desai and Mrs. Sarita Desai for financing the study..."

We note that you have provided funding information that is not currently declared in your Funding Statement. However, funding information should not appear in the Acknowledgments section or other areas of your manuscript. We will only publish funding information present in the Funding Statement section of the online submission form.

Please remove any funding-related text from the manuscript and let us know how you would like to update your Funding Statement. Currently, your Funding Statement reads as follows:

"The authors received no specific funding for this work."

Please include your amended statements within your cover letter; we will change the online submission form on your behalf.

The funding mentioned earlier was not for this study, but for raising the clinic infrastructure. That mention in the acknowledgement is removed now. So the funding statement remains unchanged.

4. We note that you have stated that you will provide repository information for your data at acceptance. Should your manuscript be accepted for publication, we will hold it until you provide the relevant accession numbers or DOIs necessary to access your data. If you wish to make changes to your Data Availability statement, please describe these changes in your cover letter and we will update your Data Availability statement to reflect the information you provide.

 Data availability statement remains unchanged and will make the repository address available on acceptance.

5. Your ethics statement should only appear in the Methods section of your manuscript. If your ethics statement is written in any section besides the Methods, please move it to the Methods section and delete it from any other section. Please ensure that your ethics statement is included in your manuscript, as the ethics statement entered into the online submission form will not be published alongside your manuscript.

 Rechecked and found appropriate.

6. Please include captions for your Supporting Information files at the end of your manuscript, and update any in-text citations to match accordingly. Please see our Supporting Information guidelines for more information: http://journals.plos.org/plosone/s/supporting-information.

5. Review Comments to the Author

Please use the space provided to explain your answers to the questions above. You may also include additional comments for the author, including concerns about dual publication, research ethics, or publication ethics. (Please upload your review as an attachment if it exceeds 20,000 characters)

Reviewer #1: Abstract
- No comments

Introduction
- The first sentence is unclear. What is the main point?

- The argument is not clearly set up. It seems that the authors argue in the first paragraph that a causal relationship between obesity and T2DM does not exist. The shift in the second paragraph to measuring fitness is abrupt.

The opening paragraph is reorganized in the revised MS for greater clarity.

- The authors contend that anthropometric measures of obesity are not as relevant as fitness, but no papers discussing how obesity relates to anthropometric measures are discussed in the introduction. It would be helpful for the authors to cite the research measuring anthropometrics in adults with obesity to explain why they feel that fitness is a more relevant measure (see below).

We have added a new paragraph in the introduction section with references to discuss the association and possible causation links between fat distribution, anthropometry and metabolic effects.

o The association of waist circumference with walking difficulty among adults with or at risk of knee osteoarthritis: the Osteoarthritis Initiative
SV Gill et al., Osteoarthritis and cartilage 25 (1), 60-66

Thanks for bringing this paper to our notice. Citing a few other papers along with this we have added a para in the discussion section that discusses the association between anthropometric parameters and functional fitness.

- Overall, the introduction needs to be reworked to clearly state what the argument is.

We hope in the revised and rewritten form the introduction has enhanced clarity and makes the purpose of the study clear.

Method
- What do the authors mean by abdominal plasticity?
- Although the authors state what areas of fitness the HRPF measures, they do not provide any details about what the 15 tasks actually are.

All details of the 15 tasks including abdominal plasticity are described in details in the supplementary information, which is cited in the main text in the methods section. The reviewer may have missed it.
-

Results
- No comments

Discussion
- A significant limitation of this study is that there is no measure of percent body fat. WHR provides a rough estimate of the distribution of body fat, but a formal measure of body fat, skeletal muscle mass, etc. would provide a clearer picture about why the authors argue the limited role of anthropometric measures in understanding limitations related to obesity.

In the paper describing the retrospective part of the study, data on body composition was not available. This can be included in a prospective study. Nevertheless we have added in the revised paper along with selected references a brief discussion on whether body fat is a better predictor of insulin resistance or T2DM.
